# Antibody production by *in vivo* RNA transfection

**DOI:** 10.1038/s41598-017-11399-3

**Published:** 2017-09-07

**Authors:** Bizhan Romani, Amirarsalan Kavyanifard, Elham Allahbakhshi

**Affiliations:** 1 Cellular and Molecular Research Center (CMRC), Faculty of Medicine, Ahvaz Jundishapur University of Medical Sciences (AJUMS), Ahvaz, 61357-15794 Iran; 2 Department of Biology, Faculty of Science, University of Isfahan, Isfahan, 81746-73441 Iran; 3 Department of Biology, Payam Noor University, Tehran, 1395-4697 Iran

## Abstract

Monoclonal antibodies have a variety of applications in research and medicine. Here, we report development of a new method for production of monoclonal antibodies. Our method relies on *in vivo* RNA transfection rather than peptide vaccination. We took advantage of RNA transcripts complexed with DOTMA and DOPE lipids to transfect mice. Intravenous administration of our RNA vaccine to mice resulted in expression of the antigenic peptides by splenic dendritic cells and detection of the antigens in the serum. The RNA vaccine stimulated production of specific antibodies against the RNA-encoded peptides. We produced monoclonal antibodies against viral, bacterial, and human antigens. In addition, we showed that our RNA vaccine stimulated humoral immunity and rescued mice infected with influenza A virus. Our method could be used as an efficient tool to generate monoclonal antibodies and to stimulate humoral immunity for research and medical purposes.

## Introduction

Human and mice have 5 antibody isotypes, which are distinguished by immunoglobulin structure^1, 2^. Currently, commercial antibodies are available in the form of monoclonal (homogenous isotype and antigen specificity) and polyclonal (heterogeneous isotype and antigen specificity) antibodies. Antibodies are essential in many biological techniques such as immunoblotting, immunoprecipitation, immunofluorescence, flow cytometry, ELISA, etc. In addition, a number of monoclonal antibodies have been approved for medical applications such as cancer therapy^3–5^. Stimulation of the immune response and antibody production is also the fundamental basis of peptide vaccines^6, 7^. The wide applications of antibodies in research and medicine demand efficient and rapid methods for antibody production.

Over the past decades, laboratory animal systems with strong humoral responses have been developed. The most common method for antibody production is based on injection of the antigenic peptides to laboratory animals in order to stimulate a humoral response^8–10^. Development of hybridoma technology was a major advance in producing large amounts of monoclonal antibodies. Using this technology, primary B cells from a vaccinated animal are fused with immortal B cells. The new hybridoma cells are then screened for production of specific antibodies^11^.

Peptide vaccines, which immunize patients against certain pathogens or cancer cells, also rely on injection of antigenic peptides^6, 12^. Similarly to monoclonal antibody production, this method requires synthesis and purification of antigenic peptides for stimulation of the humoral response. RNA transcripts, however, can be transfected *in vivo* and lead to production of substantial amounts of peptides. A recent study by Kranz *et al*. showed that using intravenously administered RNA-lipoplexes, dendritic cells can be targeted *in vivo*. Expression of the exogenous antigens resulted in activation of T cell and interferon responses^13^. In our study, we took advantage of this recently developed method for *in vivo* transfection of mice with RNA transcripts. We demonstrate production of monoclonal antibodies using RNA transfection. We show that our antibodies could be used for Western blot analysis, suggesting their potential in research. In addition, we show that our method can be used for stimulation of humoral immunity.

We report a rapid method for generation of monoclonal antibodies with potential applications in research and medicine.

## Results

### *In vitro* synthesis of RNA transcripts

**Figure 1:**
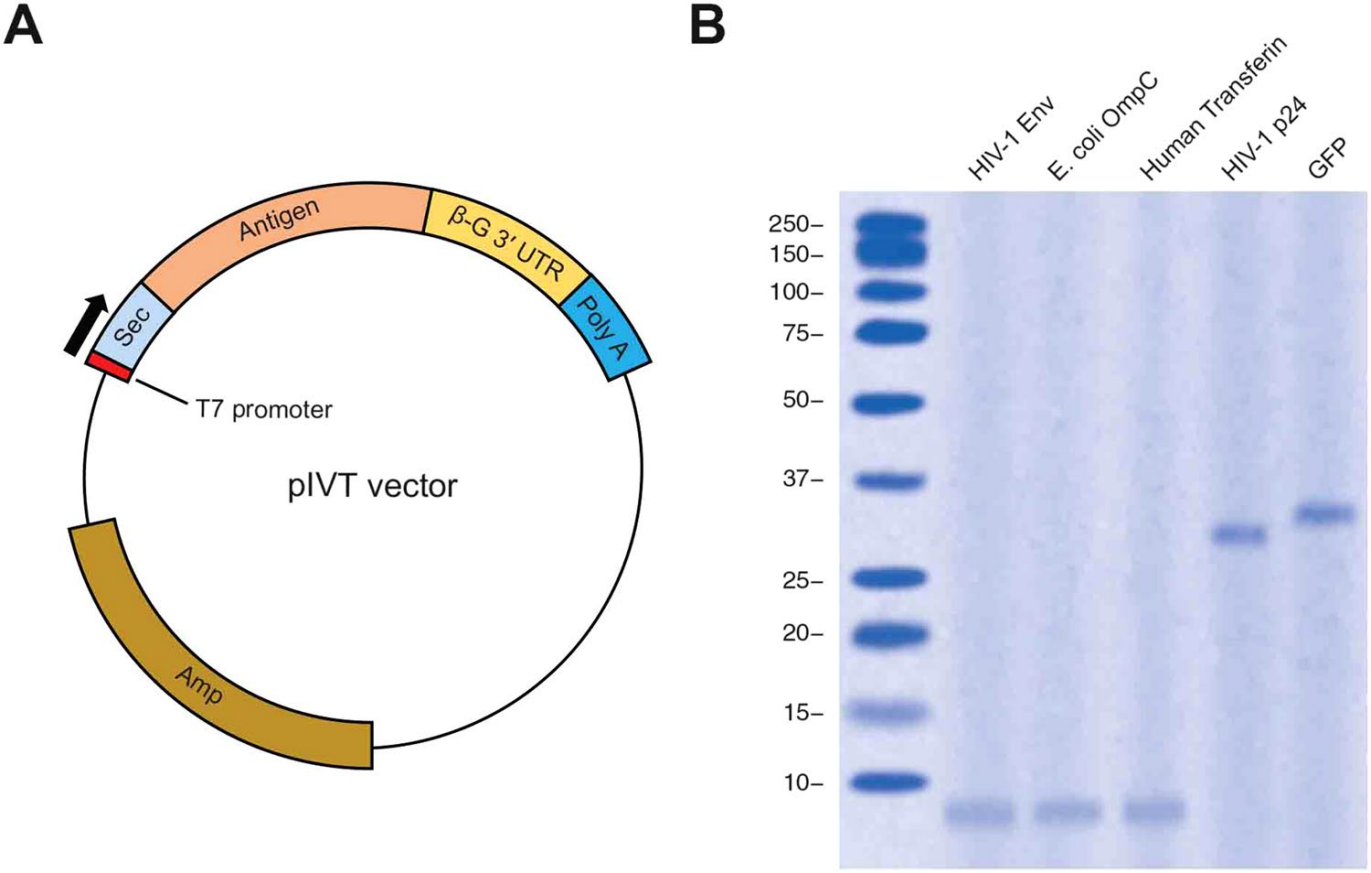
Transcription and translation of the *in vitro* transcription vector. (**A**) Schematic presentation of the pIVT vector engineered for *in vitro* transcription. (**B**) pVIT derived vectors were linearized and transcribed *in vitro*. mRNAs were translated *in vitro* and loaded on SDS-PAGE. Proteins were stained using the Coomassie blue method.

In order to generate RNA transcripts for *in vivo* transfection, we generated a construct carrying the secretory sequence the MHC class I located before the antigen sequence insert. Beta globulin 3ʹ UTR and a poly A tail were inserted after the antigen sequence for the stability of the transcripts (Fig. 1A). We cloned inserts encoding antigenic peptides from different biological sources, such as viral protein HIV-1 Env, bacterial protein OmpC, and human transferrin.

To prepare RNA transcripts for *in vivo* transfection, the constructs for each antigen were linearized using restriction enzymes and subjected to *in vitro* transcription. Transcripts were capped on their 5′-hydroxyl group. *In vitro* translation of the transcripts produced peptide fragments at expected lengths (Fig. 1B).

### Uptake and translation of RNA transcripts by dendritic cells

**Figure 2:**
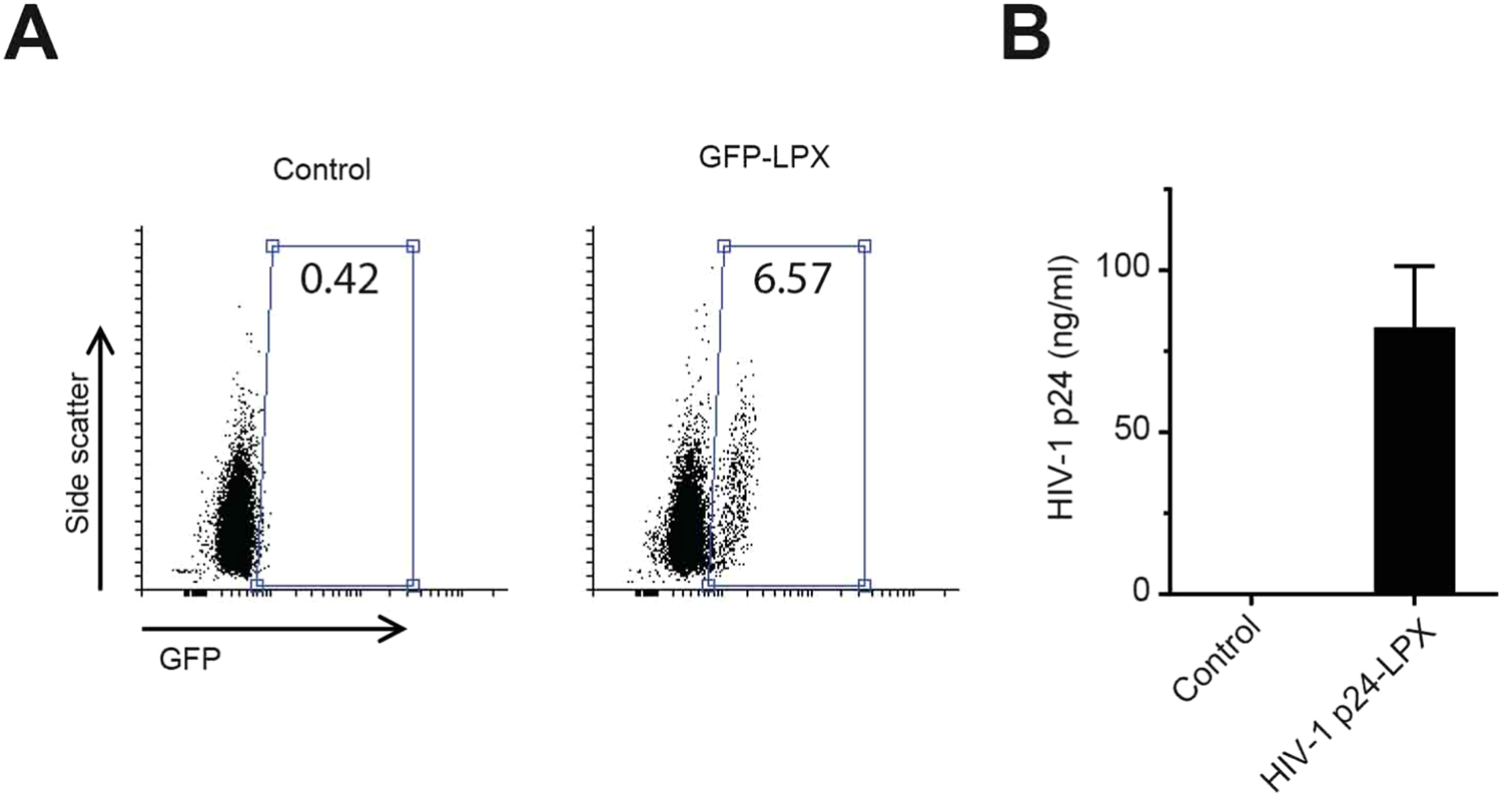
Dendritic cells are transfected by *in vitro* transcribed RNA/LPX and express RNA-encoded antigens. (**A**) BALB/c mice were injected with GFP RNA-LPX. As control, a mouse was injected with RNA-LPX. After 24 h, dendritic cells were extracted from spleens and analyzed for expression of GFP. The experiment was repeated two times and one representative experiment is shown (**B**) BALB/c mice were injected with HIV-1 p24-LPX, which expresses full length HIV-1 p24, or the RNA-LPX control (n = 3). After 24 h, blood samples were collected and HIV-1 p24 levels were measured using ELISA.

Our *in vitro* experiments demonstrated that transcription of the digested plasmids resulted in functional transcripts that were translated into peptides at expected sizes. We then tested whether these transcripts could be transfected *in vitro* to produce secretory peptides. DOTMA/DOPE liposomes were previously shown to protect RNA from ribonuclease digestion and mediated efficient uptake of the RNA transcripts mainly by dendritic cells. Uptake of the RNA transcripts will then result in expression of the encoded antigen by dendritic cells^13^. In our study, we digested a plasmid for expression of secretory GFP and transcribed it *in vitro*. The RNA copies were then mixed with DOTMA/DOPE liposomes (RNA-LPX) by adjusting the optimal net charge. The RNA-LPX was then administrated intravenously to BALB/c mice. After 24 h, expression of GFP was seen in ~6.5% of the dendritic cells isolated from spleens (Fig. 2A).

In order to stimulate a strong humoral response, antigens have to be secreted from the transfected cells. To test whether the *in vivo* produced antigens are secreted into the blood stream, we injected mice with RNA-LPX for expression of HIV-1 capsid protein (p24) tagged with the MHC-I secretory peptide. Analysis of the serum from the transfected mice indicated that p24 was detectable in the mice (Fig. 2B), suggesting secretion of p24 from the transfected cells.

### Antibody production by *in vivo* transfection of RNA transcripts

**Figure 3:**
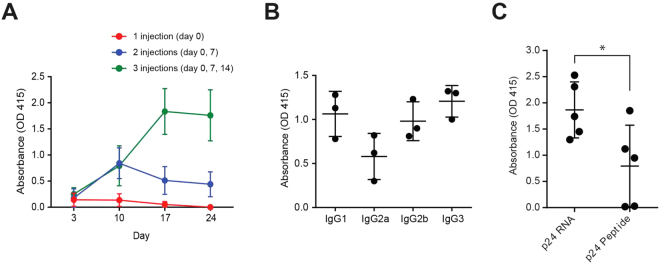
Optimization of the RNA/LPX transfections for antibody production. (**A**) BALB/c mice were transfected with p24 RNA-LPX 1, 2, or 3 times with one week intervals (n = 3 for each set of injections). Production of anti-p24 antibodies was measured on the indicated days using ELISA. (**B**) BALB/c mice that received 3 injections (**A**) were bled on day 24 and their sera were subjected to ELISA for detection of p24-specific antibody isotypes. (**C**) Five BALB/c mice were transfected with p24 RNA-LPX three times with one week intervals. Five BALB/c mice were immunized with p24 peptides three times with one week intervals. Levels of anti-p24 antibodies were measured on day 17 (3 days after the last immunization).

We showed that intravenous administration of RNA-LPX transfects dendritic cells and results in secretion of the antigenic peptide, p24. In order to examine whether our method stimulates antibody production *in vivo*, we transfected mice 1, 2, or 3 times with RNA-LPX for expression of p24. Mice were then examined for production of specific antibodies against p24 (Fig. 3A). When mice were transfected once with RNA-LPX, small amounts of anti-p24 antibodies were detectable in their serum. Double transfection of the mice resulted in about 10-fold increase in the amounts of the produced antibodies after 10 days. However, the antibody levels were not stable and ~50% reduction was observed after 1 week (day 17). Triple transfection of the mice, resulted in a significant production of anti-p24 antibodies after 17 days. After reaching the maximum levels (day n17), the antibody levels remained stable as measured on day 24. Comparison of triple transfection with double transfection indicated that triple transfection resulted in about 3.5-fold increase in the amount of the anti-p24 antibodies.

We found that triple transfection of the mice, efficiently stimulated antibody production. Triple transfection was then selected for optimal antibody production in the consequent experiments. We triple-transfected the mice with RNA-LPX for expression of p24 and examined isotypes of the produced antibodies (Fig. 3B). In our analysis, we included IgG1, IgG2a, IgG2b, and IgG3. Our analysis indicated that all the four tested antibody isotypes were produced after 24 days.

To compare the efficiency of our method with traditional peptide vaccination, we vaccinated mice with p24 RNA-LPX or p24 peptides (Fig. 3C). All 5 mice transfected with p24 RNA-LPX produced antibodies against p24. Using peptide vaccination, only 3 out of 5 immunized mice produced anti-p24 antibodies. In our comparison, we found that RNA transfection was more efficient than the traditional peptide vaccination for stimulation of antibody production.

### Specificity of monoclonal antibodies produced by *in vivo* transfection of RNA transcripts

**Figure 4:**
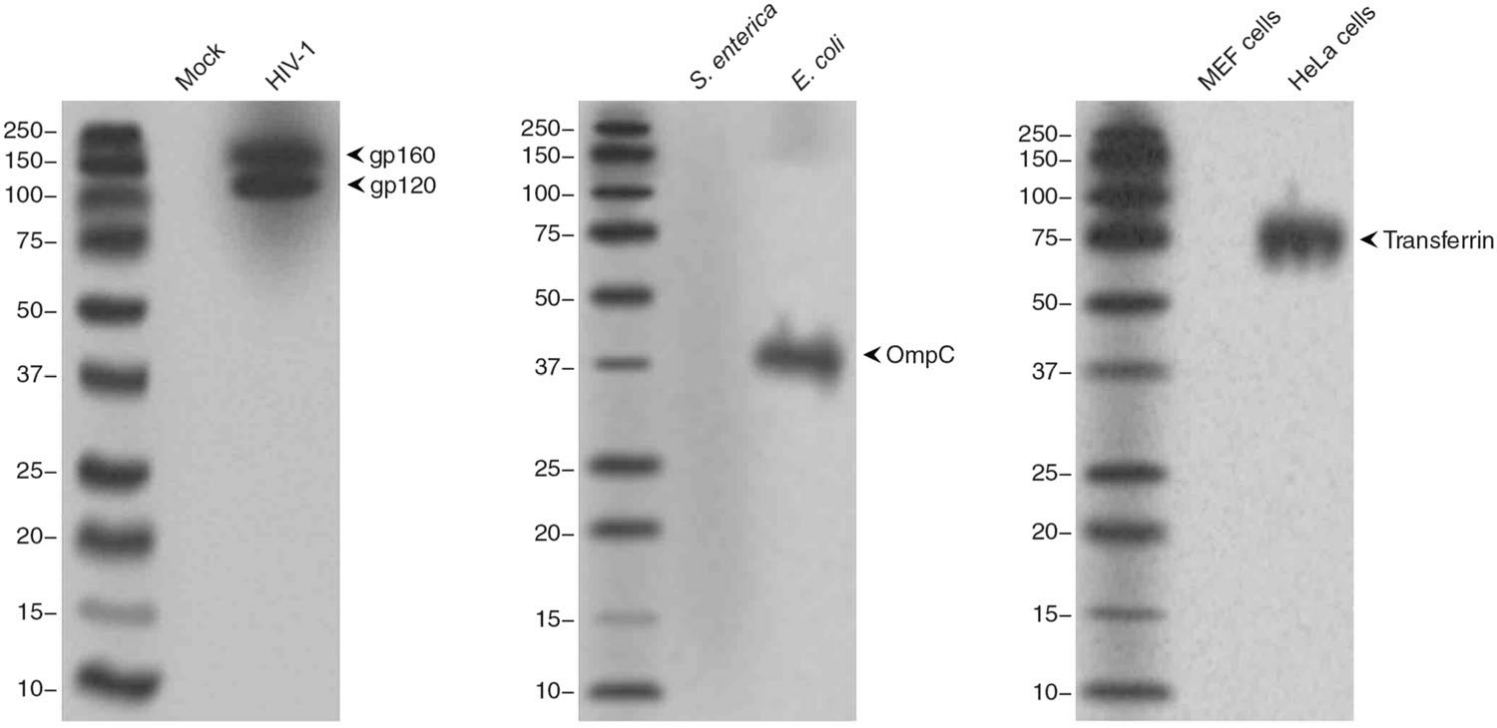
Production of monoclonal antibodies by RNA/LPX transfection. BALB/c mice were triple transfected with RNA-LPX expressing 20 amino acid fragments of HIV-1 envelope, *E*. *coli* outer OmpC, and human transferrin. On day 17, spleen cells were isolated and fused with myeloma cells to generate hybridoma cells. Monoclonal antibodies were produced by the hybridoma cells and used in Western blot analysis. To test anti-HIV-1 envelope antibody, cell lysates of HIV-1-infected and mock infected MT4 cells were analyzed (left panel). To test anti-*E*. *coli* OmpC antibody, *E*. *coli* and *Salmonella enterica* cells were lysed and analyzed (middle panel). To test anti-human transferrin, lysates of HeLa cells and mouse embryonic fibroblasts were analyzed (right panel).

Using *in vivo* transfection of RNA-LPX, we were able to stimulate production of polyclonal anti-p24 antibodies. In order to examine the utility of our method for production of monoclonal antibody against a broad range of antigens, we cloned short fragments for expression of 20 amino acid residues of HIV-1 gp160, *E*. *coli* OmpC, and human transferrin. The antigens were selected such that they included antigens of viral, bacterial and eukaryotic origins. The vectors were digested and the antigen-encoding inserts were transcribed *in vitro*. The RNA transcripts were mixed with lipoplexes and used for *in vivo* transfection. Mice were transfected with RNA-LPX three times according to our optimized method. After 17 days, spleen cells were isolated from the transfected mice and used to generate hybrid cells. Positive hybrid clones were used for production of monoclonal antibodies against the 3 aforementioned antigens (Fig. 4). Analysis of the monoclonal antibodies using Western blot demonstrated that all the antibodies detected their antigens specifically at the expected molecular weight.

### Immunization by *in vivo* transfection of RNA transcripts

**Figure 5:**
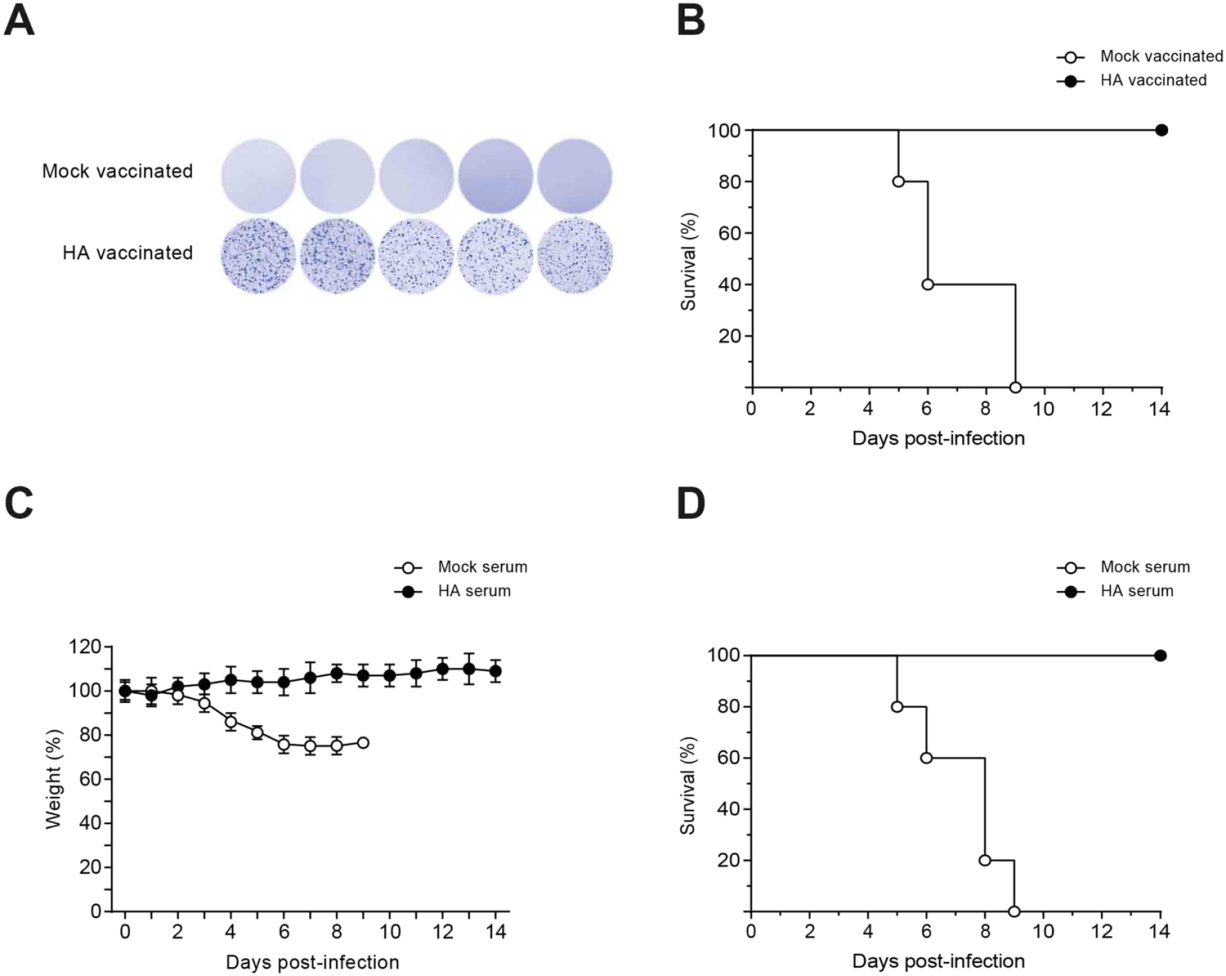
Production of anti hemagglutinin antibody by RNA/LPX confers immunity to influenza virus. BALB/c mice were transfected with RNA/LPX for expression of influenza hemagglutinin antigen or the control RNA/LPX (5 mice in each group). Transfection was repeated 2 more times with one week intervals. Three days after the last transfection (day 24) whole blood samples were collected from the mice. Antigen-specific B cell response was determined by restimulation with hemagglutinin peptides and shown by ELISPOT (**A**). The vaccinated mice were infected with lethal doses of influenza A virus and survival rate was monitored in them (**B**). A lethal dose of influenza A virus was treated with the serum of vaccinated mice (before infection) at room temperature for 30 min. Two groups of naïve BALB/c mice (n = 5 per group) were intranasally infected with serum-treated viruses. The infected mice were weighed (**C**) and survival rate was monitored (**D**) on daily basis for two weeks.

Using *in vivo* transfection of mice, we stimulated antibody production and isolated monoclonal antibodies. From an industrial or biotechnological perspective, monoclonal antibodies are invaluable. However, stimulation of humoral immunity is also a valuable strategy in immunization. We decided to examine efficiency of our *in vivo* transfection method for conferring humoral immunity. We chose influenza A virus to test our method of vaccination. Mice were intravenously injected with RNA-LPX for expression of HA antigen. Analyzing PBMCs of the vaccinated mice showed that the mice had developed B cells specifically producing anti-HA antibodies when stimulated with HA antigens (Fig. 5A). Furthermore, all the HA-vaccinated mice survived the infection while the mock vaccinated mice were euthanized 5 to 9 days post-infection due to severe weight loss (Fig. 5B).

Immunization by expression of HA antigen resulted in antibody production and conferred immunity to the vaccinated mice. However, it was not clear whether the immunity was resulted from cellular or humoral immunity. We therefore examined whether the produced antibodies in the vaccinated mice are capable of protecting the mice from viral infection. We vaccinated mice as before for expression of HA antigen. Serum of the vaccinated mice was collected and used to treat lethal doses of influenza A virus. The serum-treated viruses were used to infect the mice. Interestingly, the mice that received the HA-serum treated viruses did not lose weight and no death occurred among them. On the other hand, all the mock-serum treated mice were euthanized when they lost more than 25% of their weight (Fig. 5C and D). The experiment suggested that the antibodies raised by *in vivo* transfection are sufficient to protect mice against influenza virus infection.

## Discussion

Currently, the most widely applied method for stimulation of humoral immunity is accomplished by peptide injection, known as peptide vaccination. The method is routinely used for production of monoclonal antibodies and immunization. However, peptide vaccination has limitations such as requirement for co-injection with adjuvants, low efficiency in some cases, and requirement for high amounts of purified peptides. To date, DNA and RNA vaccines have been proposed as alternative methods to peptide vaccines^14, 15^.

In the early 1990s, the first DNA vaccines were developed by delivering plasmid DNA into the skin or muscle. The DNA vaccines induced antibody responses to viral and nonviral antigens^15–17^. Around the same time, preclinical studies of mRNA vaccines were initiated^18–22^. DNA and RNA vaccines quickly gained attention since they could stimulate broad immune responses, similarly to peptide or live-attenuated vaccines, without replicating the pathogen^15, 22^.

Acsadi *et al*. showed that intramuscular injection of DNA constructs to mice resulted in expression of human dystrophin^23^. Ulmer *et al*. injected mice with a DNA encoding the influenza virus nucleoprotein. The DNA vaccination induced generation of nucleoprotein-specific cytotoxic T lymphocytes. In addition, it prevented infection with a heterologous strain of influenza A virus. To date, DNA vaccines have been used against tuberculosis^24^, HIV^25^, anthrax^26^, malaria^27^, dengue virus^28^, typhoid^29^, etc.

Injection of DNA alone, however, is not always very immunogenic. Kim *et al*. demonstrated that DNA vaccination with α-galactosylceramide at prime phase improves immunity after boosting with antigen-expressing dendritic cells^30^. Several studies have attempted to improve the immunogenicity of the DNA vaccines by enhancing uptake of the DNA vector. Tavri *et al*. pretransfected stem cells *in vitro* with DNA-carrying microbubbles and injected mice with the pretransfected cells. The stem cells did not express the transfected GFP plasmid at this stage. The mice were then exposed to ultrasonic pulses and analysis of the mice displayed the GFP expression^31^.

Fortune *et al*. demonstrated that by enhancing hydrophobicity of the polycation, the efficiency of gene delivery in mice can be improved. They observed an enhanced expression of luciferase from a plasmid containing the luciferase gene. However, the enhancement in gene expression occurred without affecting the plasmid biodistribution. The study found that the enhanced hydrophobicity did not alter the cellular uptake but rather enhanced transfection subsequent to cellular uptake^32^.

The fate of episomal vectors for gene expression in mammals has not been well addressed. In theory, episomal DNA can integrate into the host DNA and perpetuates itself. This raises the concern for development of cancer cells. Injection of DNA could also induce antibody production against DNA^33^. On the other hand, RNA is less stable and after a limited amount of expression, RNA is degraded with no risk of integration into the host DNA. In this study, we took advantage of a recently developed protocol by Kranz *et al*. for RNA delivery to dendritic cells. It was previously shown that near-neutral and slightly negative RNA-lipoplex particles efficiently target dendritic cells *in vivo* ^13^. We applied the same method and obtained 6.57% transfection of the splenic dendritic cells. The dendritic cells expressed the transfected proteins and the secreted proteins were detectable in mouse serum. Expression of the transfected antigens induced production of different antibody isotypes, suggesting that the method can be used to stimulate production of different antibody isotypes for medical and research purposes. Furthermore, we showed that the produced antibodies can be used for Western blot analysis. Comparison of our method with peptide immunization, showed that our method stimulates production of higher amounts of antibodies in the transfected mice. In addition, our method stimulated antibody production in all the immunized animals. Due to its efficiency, our method requires sacrificing fewer animals.

The applications of this method are not limited to research antibodies. We examined our method for conferring immunity by stimulating humoral response. Injection of mice with HA RNA-LPX resulted in B cells specifically responding to HA peptides. In addition, the vaccinated mice were immune to influenza virus infection. Since the vaccination could stimulate both cellular and humoral immunity, and therefore protecting the mice, it was not clear whether the antibodies protected the mice. We treated lethal doses of influenza virus with sera collected from the vaccinated mice. The serum-treated virus did not kill the mice, suggesting the anti-HA antibody raised in the vaccinated mice were sufficient to protect the mice. However, this does not rule out the role of cellular immunity in protecting the vaccinated mice.

Taken together, RNA transfection using lipoplex complexes is an efficient method to transfect dendritic cells *in vivo*. Transfection of dendritic cells alone is of great significance. Our protocol was successfully applied to monoclonal antibody production. We also demonstrated that RNA-lipoplex could be used as a vaccination method to raise antibodies against specific pathogenic antigens.

## Methods

### Ethics statement

This study was approved by the ethics committee of Ahvaz Jundishapur University of medical sciences. All experiments were performed in accordance with the approved guidelines and regulations, and the animal experimental protocols were approved by the institutional review boards of Ahvaz Jundishapur University of medical sciences.

### Mammalian and bacterial cells

HeLa, HEK 293 T and MT4 cells were described before^34^. MDCK cells were obtained from ATCC and maintained in DMEM supplemented with 10% FBS. Murine myeloma NSO cells were from ATCC and maintained in RPMI 1640 supplemented with 2 mM glutamine and 10% FBS. MEF cells were a kind gift from Dr. Farzad Mahjour (University of Isfahan). *S enterica* and *E*. *coli* were kindly provided by Dr. Hamid-Reza Khojasteh (University of Isfahan).

### Viruses

pNL4.3 construct expressing the wild-type HIV-1 was described before^35^. HIV-1 viral particles were produced as previously described^36^ and used to infect MT4 cells at an MOI of 1.0. Influenza virus A/PR/8/34 was obtained from ATCC and grown in MDCK cells.

### Constructs for *in vitro* transcription

pIVT vector was generated by modifying the pGEM-3Z Vector (Promega, Madison, WI) and used for *in vitro* transcription of antigen-encoding RNAs. First, a 100 bp poly A tail was inserted into the pGEM-3Z Vector using HindIII restriction sites. The 3′UTR of the β-globin flanked by PstI and SphI restriction enzyme sites was amplified from activated PBMCs using β-globin 3′ UTR-Forward: 5′-TAC CTGCAG AGC TCG CTT TCT TGC TGT CCA ATT TCT-3′) and β-globin 3′ UTR-Reverse: 5′-TAC GCATGC GCA GCA ATG AAA ATA AAT GTT TTT TAT TAG GCA-3′. The amplified β-globin 3′ UTR was then inserted before the poly A tail. To direct secretion of the cloned antigens, the MHC-I secretion signal peptide (78 bp) was amplified from activated PBMCs and inserted into the pGEM-3Z Vector using sec-EcoRI-Forward, 5′-TAC GAA TTC ATG GCC GTC ACG GCG CCC CGA ACC-3′ and sec-KpnI-Reverse, 5′-TAC GGT ACC GGA GCC CGC CCA GGT CTG GGT CAG-3′. Two blunt end restriction sites, *Eco*RV and *Bpi*I, were introduced before the T7 promoter and after the poly(A) tail using site-directed mutagenesis in order to linearize the construct.

*Knp*I and *Bam*HI site-flanked fragments were synthesized for base pairs 118 to 177 (aa 40-YGVPVWKEATTTLFCASDAK-59) of HIV-1 envelope, base pairs 757 to 816 (aa 253-AQYTQTYNATRVGSLGWANK-272) of *E*. *coli* OmpC and base pair 352 to 411 (aa 118-AVVKKDSGFQMNQLRGKKSC-137) of human transferrin. Similarly, *Knp*I and *Bam*HI site-flanked full length p24 was also amplified from HIV Gag-iGFP plasmid as described before. All the antigen encoding fragments were ligated between the MHC-I secretion signal peptide and the β-globin 3′UTR.

Full length GFP was amplified from HIV Gag-iGFP plasmid using GFP-Forward, 5′-TAC GGT ACC TCG CAG AAC TAT CCA ATT GTA-3′ and GFP-Reverse, 5′-TAC GGA TCC TCT AGA CTT GTA CAG CTC GTC-3′ and subcloned into the pIVT vector. Restriction sites on both sides of the antigen-encoding regions were removed by site directed mutagenesis to avoid presentation of undesired amino acids.

### *In vitro* transcription

The pIVT-derived constructs were linearized using *Eco*RV and *Bpi*I at 37 °C for 1 h. Digestion reactions were halted by incubation at 65 °C for 20 min prior to transcription. Digested constructs were run on 1% agarose gel electrophoresis and stained with ethidium bromide. The linear fragments of interest were excised and purified using QIAquick gel extraction kit. Capped RNA transcripts were generated using the mMessage mMachine T7 ultra kit (Thermo Fisher Scientific, USA) and purified using MEGAclear Kit (Thermofisher Scientific).

### *In vitro* translation

In order to examine quality of the antigen coding fragments, the purified capped RNA transcripts were translated *in vitro* using the Retic Lysate IVT Kit as the manufacturer’s instruction. Products of *in vitro* translation were then loaded on SDS-PAGE and subjected to electrophoresis. The gel was then stained using the Coomassie blue method.

### *In vivo* transfection

Lipofectin Transfection Reagent (Thermofisher Scientific) with a 1:1 of the cationic lipid N-[1-(2,3-dioleyloxy)propyl]-n,n,n-trimethylammonium chloride (DOTMA) and dioleoyl phophotidylethanolamine (DOPE) was used to develop liposomes by the thin film hydration method. Briefly, a stock solution of Lipofectin Transfection Reagent was resuspended in 99.5% ethanol at a concentration 10 mg/ml. Ethanol was evaporated such that a lipid film developed after 1 h. The lipid film was hydrated by adding RNase-free water and gentle shakes. The final concentration of the lipid was adjusted to approximately 6 mM and incubated at 4 °C overnight. Liposomes were filtered through polycarbonate membranes with 200 nm pores. Complexes of RNA-lipoplexes with a charge ratio of 1.3:2 (lipid:RNA) were previously shown to form stable particles targeting RNA to dendritic cells^13^. To calculate charge ratio of the RNA-LPX complexes, each lipid head group was counted as one positive charge and each RNA phosphodiester was counted as one negative charge. RNA was resuspended in HEPES-buffered solution at the final concentration of 1 mg/ml. Lipoplexes and RNA were diluted in 1.5 M NaCl with a charge ratio of 1.3:2 and the NaCl concentration was adjusted to 150 mM by adding RNase-free water. RNA transcripts of pIVT-empty were used as RNA controls.

All *in vivo* transfections were performed on BALB/c mice (The Jackson Laboratory) in strict accordance with institutional protocols. Briefly, 40 μg RNA-LPX was intravenously administrated to the mice three times unless stated otherwise.

### Peptide immunization

p24 peptides were produced by transfection of HEK 293 T cells with pIVT-p24. Secreted p24 was extracted from the supernatant. For each mice, 50 µg p24 was mixed with Sigma adjuvant system at 1:1 ratio (v/v). BALB/c mice were immunized subcutaneously on the back of their neck. After 7 and 14 days, mice were intraperitoneally immunized with 50 µg p24 diluted 1:1 with Sigma adjuvant. Mice were examined for antibody production on day 17.

### Mouse infection model

BALB/c mice were anesthetized with ketamine-xylaxine and intranasally infected with 10^5^ PFU/50 µl influenza virus A/PR/8/34. Body weight and survival were measured every day. Mice were considered to have reached the experimental end point when they lost 25% of their initial weight. The experimental end point mice were humanely euthanized.

### Generation of Hybridoma cells

One week before cell fusion, murine myeloma NSO cells were cultured with 8-azaguanine to ensure their sensitivity to HAT (hypoxanthine-aminopterin-thymidine) selection medium. Transfected mice were euthanized and their spleens were removed. Spleens were homogenized to single cell suspensions using a metal cell strainer. Single spleen cells (5 × 10^7^) were mixed with NSO cells (10^7^) and polyethylene glycol was added to the final concentration of 35%. Cells were co-centrifuged at 1100 rpm for 6 min. Supernatant was removed and cells were resuspended in DMEM and centrifuged at 1000 rpm for 5 min. Cells were then resuspended in the HAT selection medium and seeded in 96-well plates. Murine IL-6 was added to the selection medium at the final concentration of 1 ng/ml and cells were incubated at 37 °C for 10 days. The supernatant in each well was screened for production of specific antibodies using ELISA. Potential positive wells were expanded into 24-well plates such that each well contained only 1 cell.

### Enzyme-linked immunosorbent assay (ELISA)

Blood samples were collected from the vaccinated mice. Serum was collected from the whole blood by centrifugation. Levels of HIV-1 p24 in the sera were measured using HIV-1 p24 Antigen Capture assay (ABL Inc.) according to the manufacturer’s instructions. To determine specific antibody production in mice, sera were collected. Two fold serial dilutions of the sera were prepared and added to 96-well plates pre-coated with the desired peptides. After 2 h incubation at 37 °C, plates were washed 3 times and an HRP conjugated anti-mouse antibody or anti-mouse isotype specific antibodies were added to the wells. Plates were incubated at 37 °C for 2 h and then washed 3 times with PBS. ABTS (ThermoFisher Scientific) was added as the colorimetric substrate for HRP. ELISA plates were read at 415 nm.

### Flow cytometry

Transfected mice were euthanized and their spleens were filtered through a metal cell strainer. Single cells were resuspended in PBS and dendritic cells were labeled using PE anti-mouse CD11c Antibody (Biolegend). Cells were then analyzed using BD FACSCalibur. PE-positive cells were gated and expression of GFP was assessed among the gated cells.

### Western blot

Cells were lysed in Laemmli buffer, heat-denatured for 5 min, and the cell lysates were run on 12% SDS-PAGE gels. Proteins were transferred to nitrocellulose membranes (Bio-Rad) and blocked with 5% non-fat milk in Tris buffered solution with 1% Tween-20 (TBST). The membrane-bound proteins were probed with the supernatant of the hybridoma cells overnight. After thorough washes, primary antibodies were probed with HRP-conjugated anti-mouse antibodies. Membranes were washed to remove unbound secondary antibodies, and then developed using ECL substrate. Membranes were photographed by exposure to Amersham Biosciences ECL films.

### Enzyme-linked immunospot (ELISPOT) assay

Each well of PVDF membrane plates were treated with 15 µl of 35% ethanol for 30-60 seconds. To each well 50 µl of 70% ethanol was added and incubated for 1 min. Plates were then washed with PBS. HA peptides were diluted at concentration of 15 µg/ml and added to the plates (100 µl/well). Plates were incubated at 4 °C overnight. Freshly isolated PBMCs (10 cells) were added to each well. Plates were then incubated at 37 °C in a CO_2_ incubator for 24 h. Cells were washed with PBS 0.1% Tween 20 for 10 min and then washed 3 times with PBS 0.1% Tween 20. Biotinylated Goat anti-Mouse IgG (Abcam) and ExtrAvidin-Alkaline Phosphatase (Sigma-Aldrich) were added to the plates and incubated for 1 h. Plates were washed and antibody production was then detected by adding BCIP/NBT substrate (Sigma-Aldrich). Plates were analyzed using ImmunoSpot S6 Macro.

### Statistical analysis

Student *t* test was performed using GraphPad Prism 6.0. A value of p < 0.05 was considered statistically significant and indicated with an asterisk (*).
